# Demonstrating the value for money of implementing evidence-based treatment: the case for further investment in magnesium sulphate as a neuroprotectant for preterm births

**DOI:** 10.3389/frhs.2025.1655385

**Published:** 2026-01-12

**Authors:** Carlos Sillero-Rejon, Hannah B. Edwards, William Hollingworth, Brent C. Opmeer, Christalla Pithara-McKeown, Frank de Vocht, Sabi Redwood, David Odd, Karen Luyt, Hugh McLeod

**Affiliations:** 1 Population Health Sciences, Bristol Medical School, University of Bristol, Bristol, United Kingdom; 2 National Institute for Health and Care Research Applied Research Collaboration West (NIHR ARC West), University Hospitals Bristol and Weston NHS Foundation Trust, Bristol, United Kingdom; 3 Vilans, National Centre of Expertise for Long Term Care, Utrecht, Netherlands; 4 Population Medicine, Cardiff University, Cardiff, United Kingdom; 5 Neonatology, Cardiff and Vale University Health Board University Hospital of Wales, Cardiff, United Kingdom; 6 Translational Health Sciences, Bristol Medical School, University of Bristol, Bristol, United Kingdom; 7 Neonatology, St Michael’s Hospital, Bristol, United Kingdom

**Keywords:** value of implementation, implementation, quality improvement, cost-effectiveness, cerebral palsy, neurodisabilities

## Abstract

**Background:**

Effective and cost-effective treatments are not always optimally implemented. The benefit forgone due to sub-optimal implementation is often not considered or estimated. We use the economic concept of “incremental net monetary benefit” (INMB) to demonstrate how this can be valued. This approach can inform decision-making when used to estimate the value for money of potential future quality improvement (QI) programmes. We illustrate these analyses using the case of antenatal magnesium sulphate (MgSO_4_), a cost-effective treatment for the prevention of cerebral palsy in preterm births. We estimate the optimal implementation of MgSO_4_, the INMB lost due to sub-optimal implementation, and the value of future implementation initiatives to increase the use of MgSO_4_.

**Methods:**

We estimated MgSO_4_ treatment implementation for babies under 32 weeks' gestation using routine data on its uptake between 2014 and 2022 in England, Scotland, and Wales. The optimal uptake level of MgSO_4_ was estimated using clinical judgment. The societal lifetime INMB of MgSO_4_ for the prevention of cerebral palsy in preterm births was obtained from the literature. The INMB of sub-optimal implementation over time was estimated as the difference between optimal and actual uptake over time in each country. We estimated the cost-effectiveness of a hypothetical future QI programme based on different scenarios of implementation effectiveness and costs.

**Results:**

The optimal uptake of MgSO_4_ was 95%. The INMB forgone associated with sub-optimal MgSO_4_ uptake has reduced over time, as uptake has increased. However, in 2022, the societal lifetime INMB forgone was still £18.2 m in England, £3.7 m in Scotland, and £1.0 m in Wales. A future QI programme across all three countries achieving a 5% increase in MgSO_4_ uptake over one year, and costing £987,500 to implement, would be cost-effective; generating £7.5 m in INMB. Future implementation initiatives are likely to be cost-effective within a range of different implementation effectiveness and costs.

**Conclusions:**

The case of MgSO_4_ treatment for preterm birth illustrates how sub-optimal implementation of evidence-based interventions can be associated with high opportunity costs measured as INMB forgone. This approach provides valuable quantification of the value for money of future QI programmes to improve the implementation of these interventions.

## 1 Introduction

Economic evaluation assesses whether healthcare interventions represent good value for money (1). Well-established methods estimate the incremental costs and benefits of an intervention compared to an alternative, often “treatment as usual” (1). Health benefits can be measured using quality-adjusted life-years (QALYs) in cost-utility analysis, as recommended by the UK National Institute for Health and Care Excellence (NICE) (2). A broader perspective can also be taken, in which non-health outcomes (such as education and employment) are included. Regardless of the perspective taken, the judgment about whether incremental costs are justified by incremental benefits depends on context. For example, high-income countries may be willing to pay more for health gains. In some jurisdictions, this trade-off is built into policy through the adoption of an explicit cost per QALY gained willingness-to-pay (WTP) threshold (1, 3). New interventions for which the cost per QALY exceeds the threshold are deemed not cost-effective and are not implemented. Complementary to this incremental cost per QALY ratio, the incremental net monetary benefit (INMB) statistic can be used to inform decision-making. The INMB combines incremental costs, benefits (i.e., QALYs) and the WTP threshold into a summary statistic (1) to express the value of an intervention in monetary terms. For this purpose, the WTP for QALY gains is established, and the INMB is calculated as [(incremental QALY x WTP)—incremental cost]. Therefore, a positive INMB indicates that the intervention is cost-effective at the relevant cost per QALY threshold.

In addition to informing decision-making on specific healthcare interventions, economic evaluation can inform decisions about implementation strategies (4, 5). This is important because the sub-optimal implementation of cost-effective treatments leads to forgone health and other economic benefits, known as opportunity costs (1). While there is wide recognition of the importance of promoting implementation initiatives, comparatively little attention has been paid to how economic analyses can be used to inform the development of quality improvement (QI) programmes to optimise the implementation of cost-effective treatments (5, 6). For this purpose, health economics methods in implementation science can calculate the benefit lost over time due to sub-optimal implementation through the cumulative INMB forgone. For example, failing to optimally implement an intervention that offers good value for money—such as a smoking cessation treatment—results in a cumulative loss. This loss can be estimated as the total INMB foregone by all individuals who did not receive the intervention (e.g., those who go on to develop avoidable chronic conditions due to continued smoking). This measure can inform the scope for potential benefits to be gained from undertaking implementation initiatives, such as QI programmes. Moreover, the value of a future implementation initiative can be estimated as the cumulative INMB resulting from forecast increases in treatment uptake (7–13).

A practical illustration of this approach can be seen in the case of antenatal magnesium sulphate (MgSO_4_) therapy given to women at risk of preterm birth. MgSO_4_ is an effective and cost-effective treatment for the prevention of cerebral palsy (CP) in preterm delivery (14–17). Since 2015, the World Health Organisation (18) and NICE (19) have recommended the administration of MgSO_4_ in preterm deliveries as a core part of maternity care. Despite these guidelines, the adoption of MgSO_4_ has been irregular in the UK and other high-income countries (20–23). For example, in infants below 30 weeks' gestation, the UK National Neonatal Audit Programme (NNAP) reported that in 2017, only 64% of eligible women received it (24). There was also high variation in uptake between different regional networks (range 49%–78%) (24). Following the launch of the National PReCePT Programme in England in 2018 (25), the Preterm Perinatal Wellbeing Package in Scotland in 2017, and locally led QI initiatives in Wales (26) uptake increased to 86% by 2022. The variation in uptake between and within units persisted over time and suggested that the adoption of MgSO_4_ remained sub-optimal (27). There is room for improvement, especially in the case of MgSO₄, when small gaps in implementation represent large economic and health opportunity costs. The National PReCePT Programme (25) established 95% as a “stretch” target for MgSO₄ uptake. However, the feasible optimal target may also be informed by actual unit and network performance, which is shaped by contextual factors. Implementing an intervention, even when there is evidence-based and clinical practice guidelines, is complex and dependent on implementation contexts (26). Previous studies (26, 28) suggest that variation in practice may stem from how QI initiatives interact with enabling factors—such as team structure or leadership—to influence the uptake of MgSO₄.

This study has two complementary aims: first, to contribute methodologically to the use of health economics methods, including INMB, to inform resource allocation decisions for implementation initiatives; and second, to illustrate these methods by evaluating the adoption of MgSO_4_ for fetal neuroprotection in preterm births in the United Kingdom. For this purpose, using the uptake of MgSO_4_ in England, Scotland, and Wales over time, we estimate the optimal implementation level and the cost-effectiveness of a hypothetical future QI programme to promote further adoption of MgSO_4_ based on different scenarios of implementation effectiveness and implementation costs (i.e., assuming different levels of increased uptake of MgSO_4_ and different costing of the implementation strategies to achieve it).

## 2 Methods

### 2.1 Population data

We used pseudonymised patient-level data on preterm babies (born at less than 32 weeks' gestation), drawn from the UK National Neonatal Research Database (NNRD) from January 2014 to December 2022, for each nation (England, Scotland, and Wales). This period covers from before the NICE guidelines were published (2015) to the most recent available data. NNRD data is standardised, routinely collected health data. It covers all NHS maternity units in England, Scotland and Wales, so it is fully representative and generalisable. MgSO_4_ data is of high quality and completeness, with less than 1% missing data in 2022 (historically up to 5% missing in 2017) (29). To our knowledge, there have been no relevant changes in coding practice in the timeframe of this study that could be influencing observed MgSO_4_ uptake rates.

### 2.2 Cost-effectiveness of MgSO_4_ treatment

We estimated the lifetime INMB of giving MgSO_4_ vs. not, calculated as incremental QALYs multiplied by the WTP threshold, minus incremental costs (1, 30). We adopted Bickford and colleagues' results (31) on the treatment cost-effectiveness of antenatal MgSO_4_ in Canada. For the UK context, it provides a combined estimate of societal lifetime incremental savings of £19,054 and incremental QALY gains of 0.24 per baby born at less than 32 weeks' gestation; after converting the estimated costs to GBP currency and 2022 prices (see Supplementary Appendix A for calculations). The cost savings included those relating to healthcare, education, housing, and employment (31). This analysis generated an INMB of £23,918 per preterm birth when applying a WTP threshold of £20,000 per QALY gained, in line with NICE guidelines (2).

### 2.3 Optimal MgSO_4_ uptake comparative performance analysis

Perfect implementation (i.e., 100% uptake) is not achievable; for example, some women may have insufficient time between presenting at the maternity unit and giving birth to administer MgSO_4_. Estimating what optimal implementation might be clinically feasible at an aggregate level is key to framing the potential impact of future implementation initiatives. The optimal level of MgSO_4_ uptake was estimated using clinical judgement (KL, DO) informed by the comparative performance of maternity units in 2022. We used funnel plots (32) of MgSO_4_ uptake and the number of preterm babies in each maternity unit, to identify comparatively and statistically high- and low-performing units by type. In the UK, neonatal units differ from Local Neonatal Units (LNUs), Special Care Baby Units (SCBUs), to Neonatal Intensive Care Units (NICUs); the latter have a higher level of activity (i.e., a higher number of preterm births following transfer from other units). We plotted the results by Operational Delivery Network (ODN) to illustrate the range of performance at a network level. The optimal level was considered at 95% uptake; sensitivity analyses also included 90% uptake.

### 2.4 Value of MgSO_4_ implementation analysis

To estimate the value of implementing MgSO_4_ we calculated the following summary INMB statistics for each nation:

(A) The cumulative lifetime INMB of MgSO_4_ treatment for all patients *actually* treated.
(B) The cumulative lifetime INMB of MgSO_4_ treatment if *optimal* treatment of patients was achieved.
(C) The INMB lost due to *sub-optimal* implementation (i.e., B minus A).

We estimated these values monthly from January 2014 to December 2022 and compared the trends in these three statistics. To illustrate the change over time, we also calculated these values for the first (2014) and last (2022) years of the observed data. If INMB lost due to sub-optimal implementation is positive, the associated opportunity costs warrant the consideration of the use of implementation initiatives to reduce the research-to-practice gap.

#### 2.4.1 The cost-effectiveness of future hypothetical implementation initiatives

The INMB lost due to sub-optimal implementation in 2022 indicates the “value of optimal implementation”, or the upper threshold of how much could be invested in a QI programme to implement optimal uptake of MgSO_4_ in that year and be viewed as good value for money.

We then estimated the potential cost-effectiveness of a hypothetical future QI programme to further increase uptake in several different scenarios of implementation effectiveness and costs (6). We used heatmaps to visualise the cost-effectiveness of hypothetical scenarios with different levels of implementation costs and effectiveness.

Three implementation effectiveness scenarios were modelled specifically: low performance (1% absolute increase in MgSO_4_ uptake), mid-performance (5% increase), and high performance (10% increase) for two specific implementation cost scenarios (baseline and high-cost). These levels of effect align with the latest results from PReCePT (29) that showed an effect on average of 5.8% (*p* < 0.001); lower and upper values (i.e., 1% and 10%) are chosen to show the effects of small and large changes in MgSO_4_ uptake, with 5% as the midpoint.

Baseline costs were based on units' and ODNs' performance and PReCePT experience (25, 33). Contrary to the PReCePT programme, which allocated the same funding at the unit level (i.e., backfill funding) and at the ODN level (i.e., regional support), we propose allocating funding in proportion to the number of maternity units in each ODN, with more resources available to support comparatively low-performing units (Supplementary Appendices B,C). The high-cost scenario has double the baseline costs to conservatively acknowledge that, in addition to targeting funding, achieving higher levels of uptake might involve more resources than those used by the PReCePT programme.

In this analysis, we also considered the uncertainty range from the probabilistic analysis conducted by Bickford and colleagues (31). Meanwhile, heatmaps show central estimates; the specific scenarios modelled accounted for the 95% credible interval (CI) of MgSO_4_ cost-effectiveness.

#### 2.4.2 Subgroup analyses

The NNAP reports primarily focus on preterm births occurring before 30 weeks' gestation, aligning with current NICE guidelines that recommend MgSO₄ treatment for this group (27). For infants born between 30 and less than 34 weeks' gestation, however, NICE advises only that treatment with MgSO₄ be “considered” (19). Despite this more cautious recommendation, economic evaluations indicate that MgSO₄ remains cost-effective for babies born before 32 weeks' gestation (14, 31). Given these clinical and economic considerations, our analyses were stratified into two subgroups: infants born before 30 weeks' gestation, and those born between 30 and under 32 weeks. We also conducted analyses for the combined group of all preterm births occurring before 32 weeks' gestation.

## 3 Results

### 3.1 Optimal MgSO_4_ uptake

**Figure 1:**
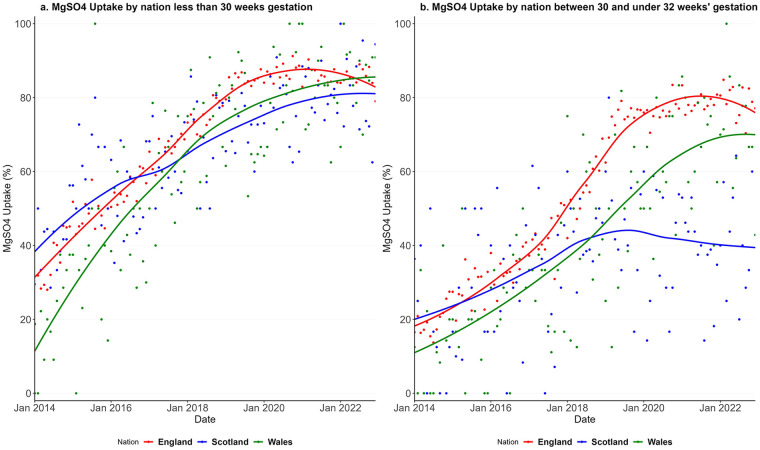
Uptake of MgSO_4_ in England, Scotland and Wales from Jan 2014 to Dec 2022 for babies less than 30 weeks' gestation **(a)** and between 30 and under 32 weeks' gestation **(b).**

Figure 1 includes the uptake of MgSO_4_ over time for the three nations for babies born with less than 30 weeks' gestation and for babies born between 30 and less than 32 weeks' gestation. MgSO_4_ uptake increased from 36% in 2014 to 85% in 2022 in England, from 39% to 82% in Scotland, and from 20% to 86% in Wales for babies born under 30 weeks' gestation (Figure 1a). Uptake increased from 19% in 2014 to 79% in 2022 in England, from 19% to 42% in Scotland, and from 13% to 72% in Wales for babies born between 30 and under 32 weeks' gestation (Figure 1b). There is comparatively more monthly variability in percentage uptake in Wales and Scotland due to the smaller number of babies compared to England. In England, a plateau in uptake and a decrease towards the end of the observed period are apparent. The uptake for babies between 30 and under 32 weeks' gestation is lower than that for babies under 30 weeks' gestation, particularly in Scotland. Overall, uptake has increased substantially over time, although Scotland lagged behind for babies born between 30 and just under 32 weeks' gestation.

**Figure 2:**
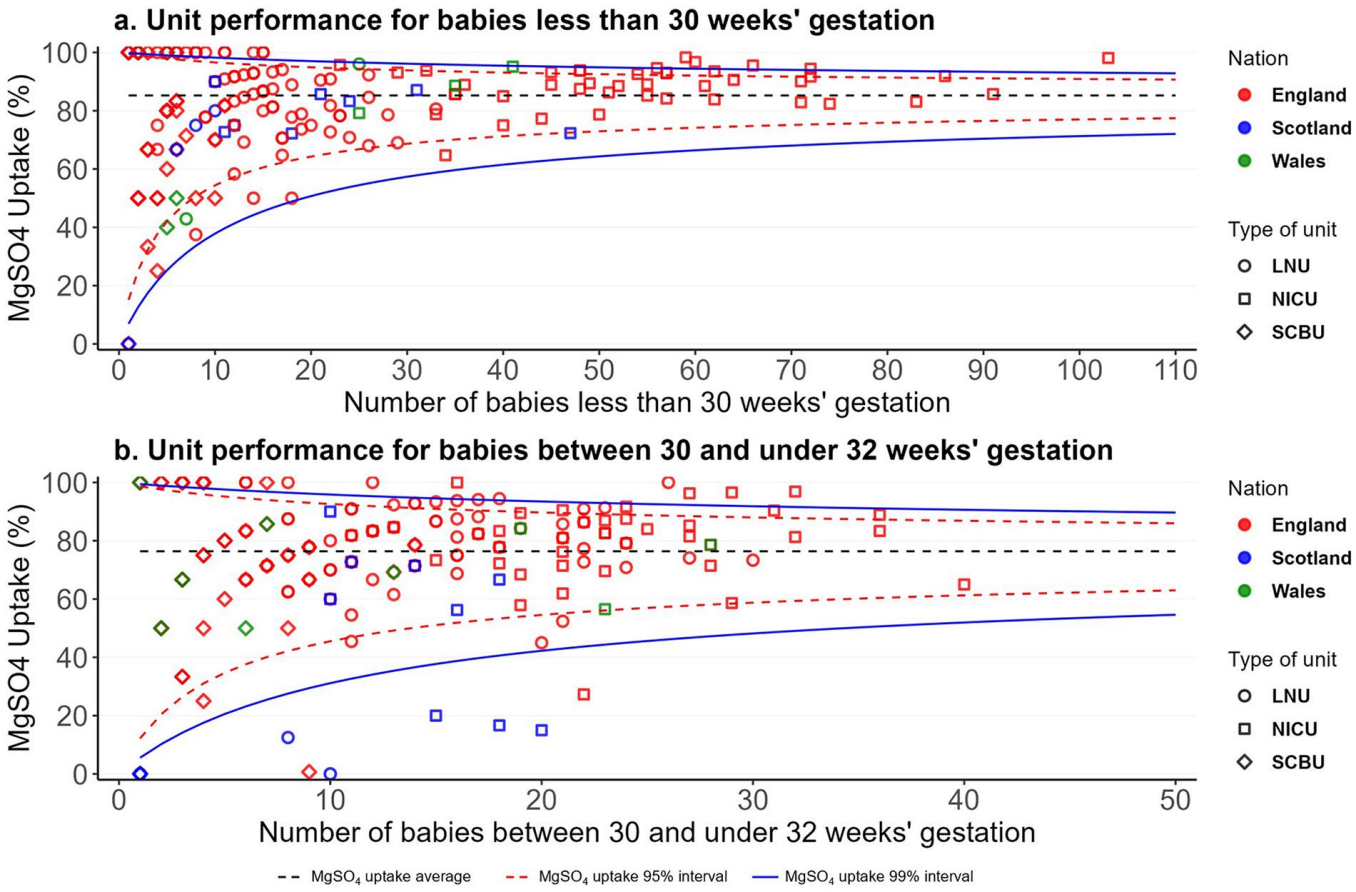
MgSO_4_ uptake unit performance in England, Scotland and Wales in 2022 for babies less than 30 weeks' gestation **(a)** and between 30 and under 32 weeks' gestation **(b)**.

Figure 2 shows the unit performance in MgSO_4_ uptake by type of unit for the three nations in funnel plots for babies born with less than 30 weeks' gestation and for babies born between 30 and less than 32 weeks' gestation. The funnel plot for babies under 30 weeks' gestation shows that in 2022, from 175 units, 43 (25%) were high-performing units with an uptake average of 97.9% (Figure 2a). 13 out of 78 (17%) of LNUs, 14 out of 43 (33%) of SCBUs, and 16 out of 54 (30%) of NICUs were high-performing units (Figure 2a). The funnel plot for babies between 30 and under 32 weeks' gestation shows that in 2022, from 175 units, 35 (20%) were high-performing units with an uptake average of 96.8% (Figure 2b). 17 out of 78 (22%) of LNUs, 10 out of 43 (23%) of SCBUs, and eight out of 54 (15%) of NICUs were high-performing units (Figure 2b). Supplementary Appendices D,E show that there was wide variation in units' performance within each clinical network (ODN) in 2022. For babies born at less than 30 weeks' gestation, performance generally improves with unit volume; smaller units show greater variability due to the rarity of preterm births. However, even among high-volume units, uptake varies widely—from below 80% to above 95% in the high-performing units. This variation implies inequitable care and negative health and societal consequences. For babies between 30 and under 32 weeks' gestation, variation is even more pronounced and appears less related to case volume, suggesting inconsistent interpretation or application of guidelines.

### 3.2 Value of MgSO_4_ implementation

21% (36/175) of units achieved at least 95% uptake in 2022, and this level of performance was considered both feasible and optimal for the value of MgSO_4_ implementation analyses.

**Figure 3:**
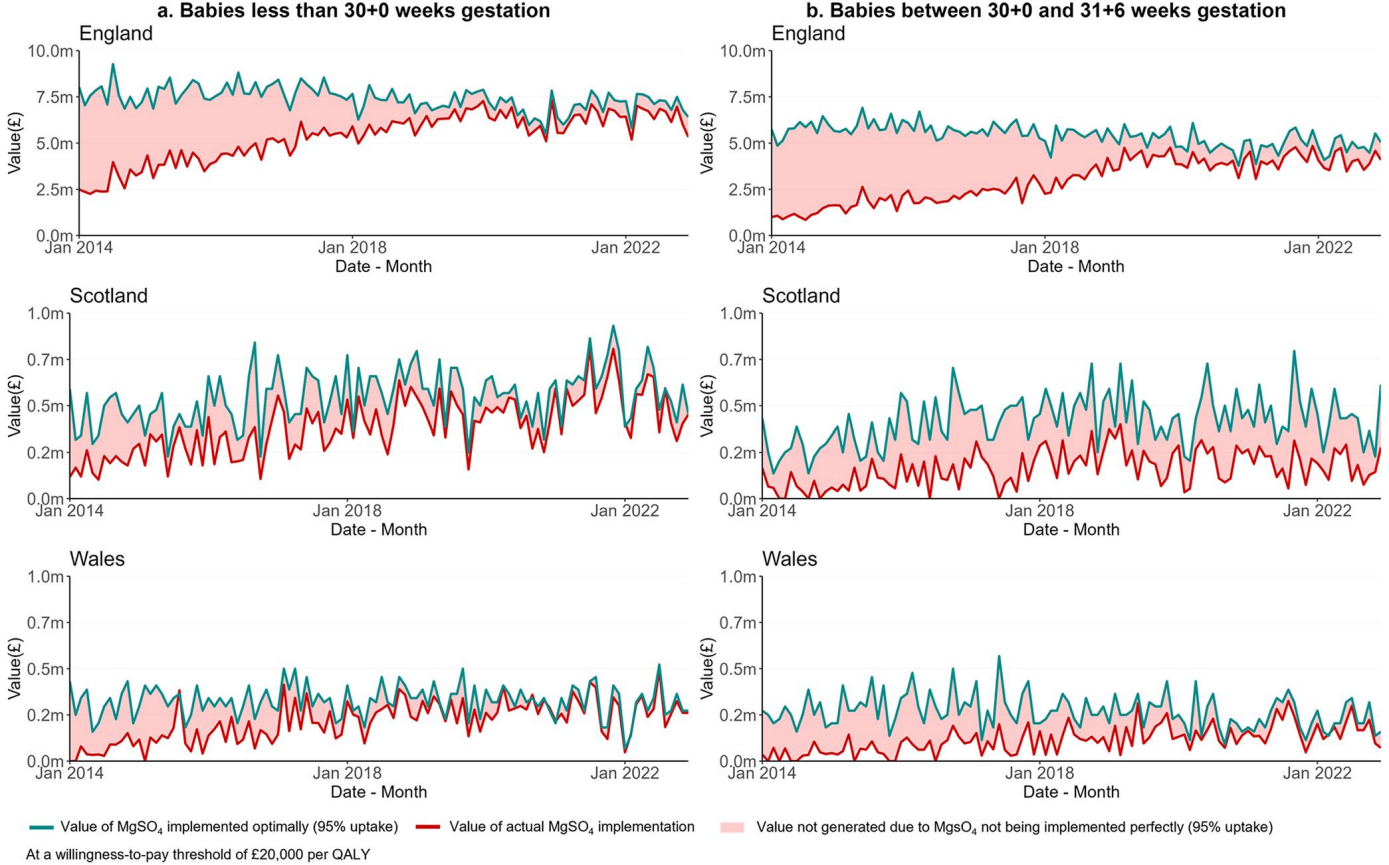
Value of optimal implementation of MgSO4 uptake in England, Scotland and Wales from Jan 2014 to Dec 2022 for babies less than 30 weeks' gestation **(a)** and between 30 and under 32 weeks' gestation **(b)**—optimal implementation considered at 95%.

Figure 3 shows the value of MgSO_4_ implementation in the three nations over time. For babies under 30 weeks' gestation in each nation, Figure 3a shows the monthly INMB of the actual implementation of MgSO_4_ (red line), the INMB of MgSO_4_ optimal implementation estimated as 95% uptake (green line), and the red area between these two lines represents the INMB lost due to sub-optimal implementation. The INMB lost (i.e., the red area) reduced progressively over time for babies born under 30 weeks' gestation (Figure 3a) and for babies between 30 and under 32 weeks' gestation (Figure 3b). This indicates that the benefits lost from not implementing MgSO₄ optimally have decreased over time as uptake has improved. However, substantial benefits are still being forgone, highlighting a persistent research-to-practice gap.

Table 1 shows the INMB of MgSO_4_ implementation for the first and last year of available data (2014 and 2022, respectively). Considering a 95% uptake as optimally, in England in 2014, the INMB generated from providing this treatment to 36% of babies of under 30 weeks' gestation (*N* = 4,003) was more than £34 m; with approximately £56 m of INMB forgone for the remaining 59% who did not, but optimally could have, received treatment (Table 1). In the same year in Scotland, the 39% uptake (*N* = 237) generated INMB of approximately £2 m, with approximately £3 m of INMB lost. Similarly, in Wales, the 20% uptake (*N* = 160) generated an INMB of £0.7 m, with around £2.9 m of INMB forgone. By 2022, the INMB forgone due to sub-optimal implementation was £8.5 m in England, £1.0 m in Scotland and £0.3 m in Wales (Table 1). The total INMB lost due to sub-optimal implementation for babies under 32 weeks' gestation in 2022 was approximately £18.2 m in England—this is equivalent to approximately 760 babies not receiving the treatment, 180 QALYs lost, and more than £14 m lifetime societal savings lost. This value equals £3.7 m in Scotland and £1.0 m in Wales (Table 1). Supplementary Appendix F shows results considering 90% as an optimal uptake.

**Table 1:** Net monetary benefit of MgSO_4_ implementation in 2014 and 2022 for England, Scotland and Wales—optimal implementation considered at 95%.

Gestation weeks	Dimensions	England		Scotland		Wales	
		2014	2022	2014	2022	2014	2022
Less than 30	Number of babies, N	4,003	3,744	237	292	160	152
	Monthly average of uptake of MgSO_4_ (%)	36%	85%	39%	82%	20%	86%
	INMB of optimal MgSO_4_ implementation (95%), £	90,956,566	85,071,542	5,385,138	6,634,853	3,635,536	3,453,759
	INMB of actual implementation, £	34,293,919	76,530,120	2,205,233	5,705,205	735,248	3,152,670
	INMB forgone due to sub-optimal implementation (95%), £	56,662,647	8,541,422	3,179,905	929,648	2,900,288	301,090
Between 30 and under 32	Number of babies, N	3,042	2,553	139	215	134	117
	Monthly average of uptake of MgSO_4_ (%)	19%	79%	19%	42%	13%	72%
	INMB of optimal MgSO_4_ implementation (95%), £	69,120,628	58,009,521	3,158,372	4,885,252	3,044,761	2,658,486
	INMB of actual implementation, £	14,072,670	48,260,861	673,111	2,148,588	431,188	2,005,685
	INMB forgone due to sub-optimal implementation (95%), £	55,047,958	9,748,660	2,485,261	2,736,663	2,613,573	652,801
Less than 32 (Total)	Number of babies, N	7,045	6,297	376	507	294	269
	Monthly average of uptake of MgSO_4_ (%)	28%	82%	29%	62%	17%	79%
	INMB of optimal MgSO_4_ implementation (95%), £	160,077,195	143,081,064	8,543,510	11,520,105	6,680,297	6,112,245
	INMB of actual implementation, £	48,366,589	124,790,981	2,878,343	7,853,794	1,166,436	5,158,354
	INMB forgone due to sub-optimal implementation (95%), £	111,710,605	18,290,083	5,665,166	3,666,311	5,513,861	953,891

MgSO_4_, magnesium sulphate.

INMB, incremental net monetary benefit, estimated at a willingness-to-pay threshold of £20,000 per quality adjusted life year.

### 3.3 Cost-effectiveness of a future implementation initiative

**Figure 4:**
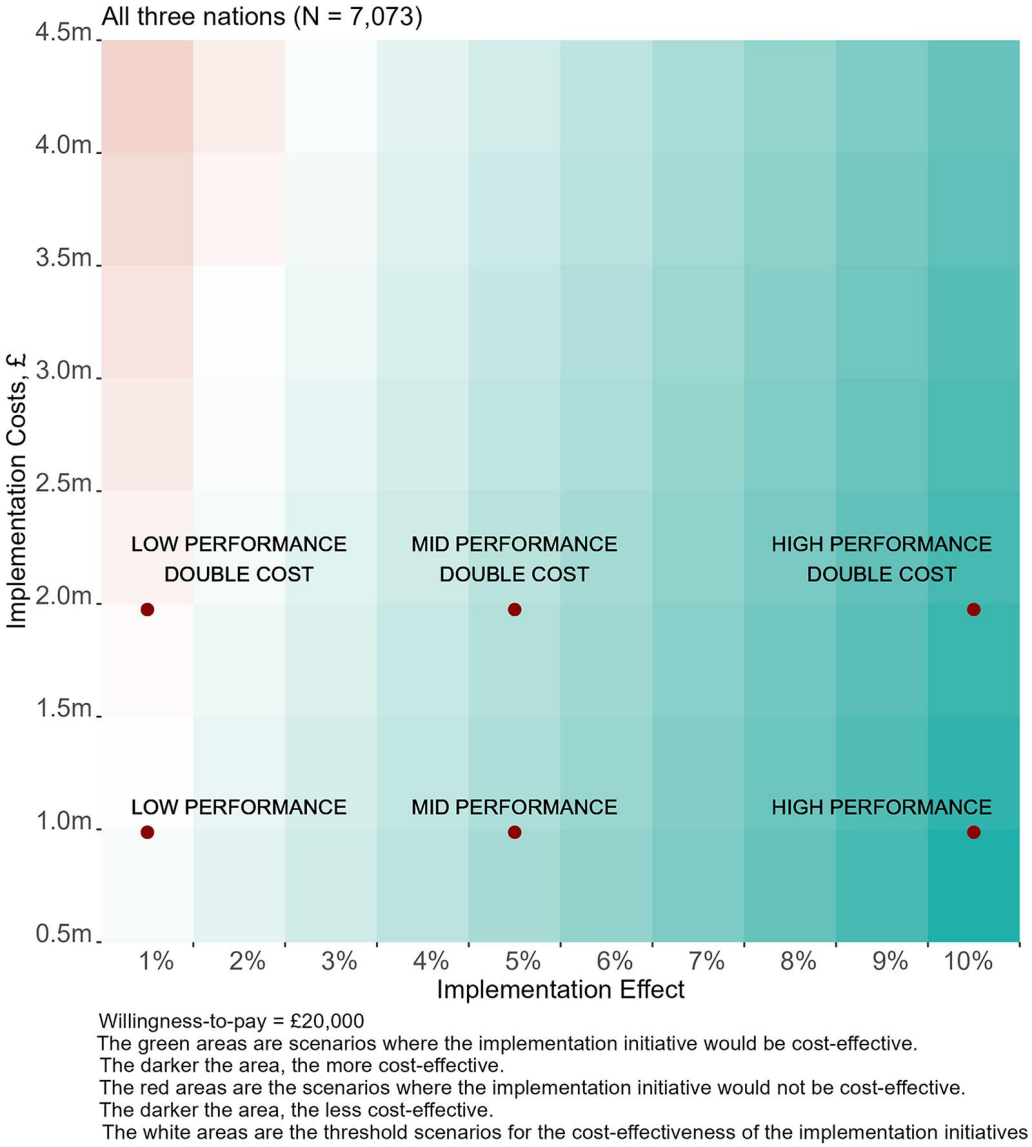
Value of implementation for potential initiatives to increase MgSO_4_ uptake in England, Scotland and Wales.

Figure 4 visualises scenarios for the cost-effectiveness of future implementation efforts over a year in the three nations for a wide range of implementation costs and effectiveness. In most of the scenarios, the hypothetical implementation initiative is cost-effective (INMB > 0), highlighting the significant scope for further QI initiatives in all three nations if they are effective in further increasing MgSO_4_ use. Baseline and high-cost scenarios are also highlighted.

Table 2 includes the results for the three specific scenarios in the baseline costs for a single-year period, also illustrated in Figure 4. Supplementary Appendix F shows the results of the high-cost scenarios. For the baseline cost, the total implementation cost would be £987,500, with the largest ODN in England receiving £102,000 and the smallest £32,500 (Supplementary Appendices B,C). If these implementation efforts resulted in, on average, a 1% increment in MgSO_4_ uptake, this would be cost-effective in England with an INMB of approximately £668,000 (95% CI from £0.2 m to £1.2 m). For Scotland (INMB £27,000, 95%CI from -£11,000 to £70,000) and Wales (INMB £10,000, 95% CI from -£10,000 to £32,000), estimates include values below zero, suggesting there is uncertainty about the cost-effectiveness of such an implementation initiative. This uncertainty is largely attributable to the smaller populations in Scotland and Wales, which increases the probability of the cumulative impact of the intervention falling short of the threshold typically required to demonstrate value for money. In practice, it would be advantageous to implement a UK-wide programme, rather than country-level interventions.

**Table 2:** National value of implementation for potential initiatives to increase MgSO_4_ uptake in England, Scotland and Wales with three different implementation effectiveness and implementation costs for a single year (under 32 weeks' gestation).

Dimensions	England	Scotland	Wales	Total
Number of babies, *N*	6,297	507	269	7,073
Low performance: Implementation effect: 1%				
Increment of pre-term babies treated with MgSO_4_	63	5	3	71
Net cost of implementation, £	838,500	94,500	54,500	987,500
Implementation cost-effectiveness, £ per additional patient treated	13,316	18,639	20,260	13,962
Net Monetary Benefit of the Policy*, £	667,671 (198,136; 1,201,499)	26,769 (−11,036; 69,750)	9,842 (−10,216; 32,646)	704,281 (176,884; 1,303,895)
Mid performance: Implementation effect: 5%				
Increment of pre-term babies treated with MgSO_4_	315	25	13	354
Net cost of implementation, £	838,500	94,500	54,500	987,500
Implementation cost-effectiveness, £ per additional patient treated	2,663	3,728	4,052	2,792
Net Monetary Benefit of the Policy*, £	6,692,353 (4,374,070; 9,284,693)	511,843 (325,188; 720,564)	267,209 (168,174; 377,950)	7,471,405 (4,867,432; 10,383,207)
High performance: Implementation effect: 10%				
Increment of pre-term babies treated with MgSO_4_	630	51	27	707
Net cost of implementation, £	838,500	94,500	54,500	987,500
Implementation cost-effectiveness, £ per additional patient treated	1,332	1,864	2,026	1,396
Net Monetary Benefit of the Policy*, £	14,223,206 (9,586,639; 19,407,885)	1,118,186 (744,875; 1,535,628)	588,917 (390,849; 810,400)	15,930,310 (10,722,364; 21,753,914)

MgSO_4_, magnesium sulphate.

INMB, incremental net monetary benefit, estimated at a willingness-to-pay threshold of £20,000 per quality adjusted life year.

* Values were calculated with a lifetime health effect of MgSO_4_ treatment per patient, Δbt, Quality Adjusted Life Year of 0.24 (0.16; 0.33) and lifetime costs MgSO_4_ treatment per patient, Δct, £ of −19,126 (−13,310; −25,648) at a willingness-to-pay threshold of £20,000 per Quality Adjusted Life Year.

Achievement of a 5% average increment of MgSO_4_ uptake would, however, be likely to be cost-effective in all three nations (Table 2) with an INMB over £6.6 m (95% CI from £4.3 m to 9.3 m) for England, over £510,000 (95% CI from 325,000 to 720,000) for Scotland and £267,000 (95% CI from 168,000 to 377,000). An implementation effort with a 10% increment of MgSO_4_ uptake would be highly cost-effective (Table 2).

## 4 Discussion

Our study demonstrates that health economic methods can effectively quantify the value of implementing evidence-based treatments and support the prioritisation of future initiatives within a budget-constrained health and care system. In the case of MgSO_4_ for preterm neuroprotection, we estimate that its implementation generated over £900 million in societal benefits between 2014 and 2022 for babies born under 32 weeks' gestation. However, sub-optimal uptake during this period resulted in forgone benefits exceeding £555 million, highlighting a significant missed opportunity in preventing CP among preterm infants. These findings underscore the need for consideration for further investment in implementation initiatives. We estimated that an additional QI programme costing £1 m to further increase adoption of MgSO_4_ would be a good use of resources (i.e., cost-effective) if it achieved at least a 1% absolute increase in uptake, and would be highly cost-effective if it achieved 5% or more additional uptake.

In England and Wales, NICE considers two key aspects of health technology implementation. First, its Health Technology Assessment Manual (2) emphasises the importance of evaluating the resource use and costs associated with implementation. It also advocates using economic evaluation evidence to estimate appropriate levels of implementation and the expected impact on the population. Second, NICE acknowledges that implementing its guidelines may require additional resources for clinicians and commissioners (34). However, it does not provide detailed methods for evaluating implementation. In the wider literature, methods including value of implementation (6, 7, 35) and policy cost-effectiveness (35, 36) have been proposed, though their application remains limited (5, 35). Our approach reconciles these methods and assesses value for money of implementation initiatives using the INMB measure (6).

We applied this approach to the case MgSO₄, recommended in NICE guidelines since 2015 for preterm neuroprotection (19). Using clinical judgement and unit-level performance data, we estimated an optimal level of implementation and combined evidence on the treatment's cost-effectiveness with the cost-effectiveness of strategies to increase uptake. Despite strong clinical consensus and policy support for perinatal optimisation, research has identified several barriers to optimal MgSO₄ uptake. These include context-specific and clinician-related factors, such as ambiguity in clinical guidance and variation in team capacity, resources, and safety culture across organisations (26, 28). Effective implementation strategies must address these challenges. Our previous work showed that a national QI programme, focused on building perinatal team capacity and capability, enabled improved uptake through a co-developed toolkit and implementation blueprint (28). However, different settings require tailored levels of QI input depending on local context (26). Therefore, we recommend that future implementation funding prioritise lower-performing units at the ODN level, where targeted support could enhance service integration and uptake. Such initiatives are likely to represent a good use of limited resources, even if they achieve only modest improvements in adoption.

Our results reveal notable differences in MgSO₄ uptake between babies born under 30 weeks' gestation and those between 30 and just under 32 weeks. In England and Wales, this may reflect how NICE guidelines are interpreted, which emphasise treatment for babies under 30 weeks, with less attention to those at higher gestational ages (19). Scotland, where these guidelines do not directly apply, showed even lower uptake for the 30–32 week group. Economic evidence supports MgSO₄ as a highly cost-effective intervention for babies under 32 weeks (14, 31). While recent reviews confirm its effectiveness up to 34 weeks (15, 37), emerging studies have introduced uncertainty about its protective effect between 30 and 34 weeks (38). This has led to the consideration of 30 weeks as a safety cut-off (39). However, given the large number of births between 30 and 32 weeks, our data suggest that the opportunity costs of non-treatment in this group are substantial. Balancing treatment costs, opportunity costs, and potential adverse effects needs to be considered in determining the most appropriate gestational age threshold. In light of recent evidence, our findings, and WHO's recommendation to treat babies under 32 weeks (18), future guidelines and QI programmes should explicitly aim to increase uptake in the 30–32 week subgroup. These differences also point to the potential value of targeted QI interventions.

Despite the important implications of our study in applying health economic methods to assess the impact of implementing evidence-based treatments, several limitations and areas for future research remain. There is scope for further methodological development, particularly for interventions that require sustained adherence (e.g., weight loss medications or psychological therapies), assess greater uncertainty between implementation and treatment outcomes, or raise equity considerations. While these methods are transferable to other settings, results are likely to be context-specific. In the case of MgSO₄, our analysis is constrained by limited evidence on its lifetime cost-effectiveness (40) and does not account for substantial clinical negligence litigation costs associated with perinatally acquired CP. More robust data on the long-term impact of CP are needed to fully understand its societal and individual burden. Additionally, our estimates rely on assumptions about optimal uptake. Although informed by clinical judgment and unit-level performance, further work—such as engagement with key informants—could refine these assumptions. Achieving higher levels of uptake may also require more intensive efforts and greater resource investment. Therefore, results from scenario analyses involving high-performance implementation (i.e., a 10% increase in uptake) should be interpreted with caution, as such levels may be difficult to achieve in practice. Finally, our analysis is limited to the lower WTP threshold of £20,000 per QALY. It is important to highlight that if higher WTP thresholds (e.g., £30,000 per QALY) are used, our conclusions would be even more strongly supported.

## 5 Conclusion

Health economic methods offer a valuable framework for informing decision-makers about the potential impact and value for money of future implementation initiatives. In our case study of antenatal magnesium sulphate for the prevention of cerebral palsy in preterm babies, we found that its sub-optimal implementation results in substantial societal benefits lost, highlighting the importance of investing in strategies to improve uptake. This approach enables the quantification of the potential value for money of future quality improvement programmes, and offers a compelling rationale for targeted investment in the implementation of magnesium sulphate.

## Data Availability

The data analyzed in this study is subject to the following licenses/restrictions: Anonymised individual-level data for this study are from the NNRD. Our data-sharing agreement with the NNRD prohibits sharing data extracts outside of the University of Bristol research team. The NNRD data dictionary is available online and copies of the Statistical analysis plan are available at request. Requests to access these datasets should be directed to https://www.imperial.ac.uk/neonatal-data-analysis-unit/neonatal-data-analysis-unit/.
