# Supplementary material for: Demonstrating the value for money of implementing evidence-based treatment: the case for further investment in magnesium sulphate as a neuroprotectant for preterm births
Source: Front Health Serv. 2026 Jan 12;5:1655385. doi: 10.3389/frhs.2025.1655385 (PMC12833438; doi:10.3389/frhs.2025.1655385)
Supplement: Supplementary file 1 [file Table1.docx]

# **Supplementary Material**

**Demonstrating the value for money of implementing evidence-based treatment: the case for further investment in magnesium sulphate as a neuroprotectant for preterm births**

Carlos Sillero-Rejon^1,2^, Hannah B Edwards^1,2^, Brent C. Opmeer^2,3^, William Hollingworth^1,2^, Christalla Pithara-McKeown^1,2^, Frank de Vocht^1,2^, Sabi Redwood^1,2^, David Odd^4,5^, Karen Luyt^1,2,6,7^, Hugh McLeod^1,2^

**Corresponding author**: Carlos Sillero-Rejon

# **Appendix A.** Estimated lifetime costs and QALYs per patient associated with MgSO_4_ treatment (2022 prices).

| **Type of birth** | **Perspective** | **MgSO_4_** | **Cost, £** | **Δcost, £** | **QALYs** | **ΔQALYs** |
| --- | --- | --- | --- | --- | --- | --- |
| Imminent | Societal | Yes | 62,175 | -23,768 | 26.6 | 0.3 |
|  |  | No | 85,944 |  | 26.3 |  |
| Threatened | Societal | Yes | 44,213 | -16,017 | 26.7 | 0.2 |
|  |  | No | 60,230 |  | 26.5 |  |
| Combined (40% imminent) | Societal | Yes | 51,398 | -19,118 | 26.7 | 0.24 |
|  |  | No | 70,515 |  | 26.4 |  |

Based on Bickford et al. [29]

UK prices were calculated in two steps: first, converting the cost estimates from Can$ to GBP using exchange rates. Second, adjusting the original cost estimates from the original price year to a target price year (2022), using a Gross Domestic Product deflator index obtained from the International Monetary Fund.

# **Appendix B.** Illustrative hypothetical unit-level funding for a future Quality Improvement programme: baseline and high cost scenarios.

| Unit type | Unit-level funding by 2022 performance rating (£) | | | | | |
| --- | --- | --- | --- | --- | --- | --- |
|  | baseline scenario | | | high cost scenario | | |
|  | standard | high | low | standard | high | low |
| LNU | 4,000 | 2,000 | 6,000 | 8,000 | 4,000 | 12,000 |
| NICU | 7,500 | 5,500 | 9,500 | 15,000 | 11,000 | 19,000 |
| SCBU | 7,500 | 5,500 | 9,500 | 15,000 | 11,000 | 19,000 |

# **Appendix C.** Illustrative hypothetical Operational Delivery Network and national funding for a future Quality Improvement programme.

| **Nation** | **English ODNs** | **Number of maternity units by performance level in 2022** | | | | **funding scenario (£)** | |
| --- | --- | --- | --- | --- | --- | --- | --- |
|  |  | **standard** | **high** | **low** | **total** | **baseline** | **high cost** |
|  | East Midlands | 9 | 0 | 1 | 10 | 59,500 | 119,000 |
|  | East of England Perinatal | 15 | 2 | 0 | 17 | 88,500 | 177,000 |
|  | Kent, Surrey, Sussex | 11 | 1 | 1 | 13 | 87,000 | 174,000 |
|  | London - North Central & East | 7 | 2 | 1 | 10 | 52,000 | 104,000 |
|  | London - North West | 5 | 1 | 0 | 6 | 32,500 | 65,000 |
|  | London - South | 6 | 3 | 1 | 10 | 53,500 | 107,000 |
|  | North West | 13 | 5 | 2 | 20 | 102,000 | 204,000 |
|  | Northern | 9 | 1 | 0 | 10 | 73,000 | 146,000 |
|  | South West | 9 | 3 | 0 | 12 | 63,000 | 126,000 |
|  | Thames Valley & Wessex | 9 | 4 | 0 | 13 | 58,000 | 116,000 |
|  | West Midlands | 9 | 5 | 0 | 14 | 77,500 | 155,000 |
|  | Yorkshire & Humber | 13 | 3 | 1 | 17 | 92,000 | 184,000 |
| England |  | 115 | 30 | 7 | 152 | 838,500 | 1,677,000 |
| Scotland |  | 10 | 2 | 2 | 14 | 94,500 | 189,000 |
| Wales |  | 4 | 4 | 1 | 9 | 54,500 | 109,000 |
| Total |  | 129 | 36 | 10 | 175 | 987,500 | 1,975,000 |

**Appendix D.** MgSO_4_ uptake unit performance in England, Scotland and Wales in 2022 by Operational Delivery Network for babies under 30 weeks’ gestation.

**
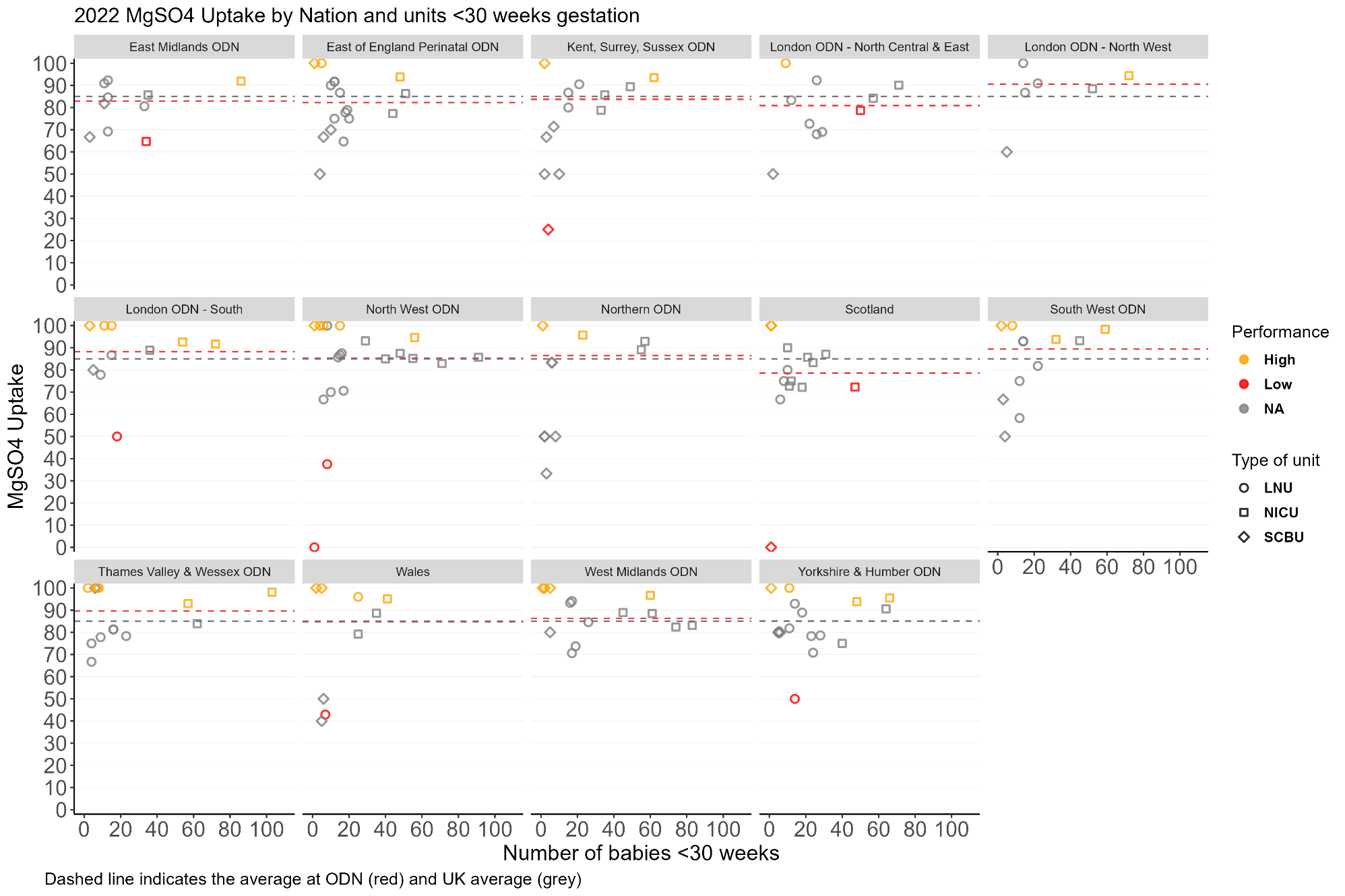
**

**Appendix E.** MgSO_4_ uptake unit performance in England, Scotland and Wales in 2022 by Operational Delivery Network for babies between 30 weeks and under 32 weeks’ gestation.


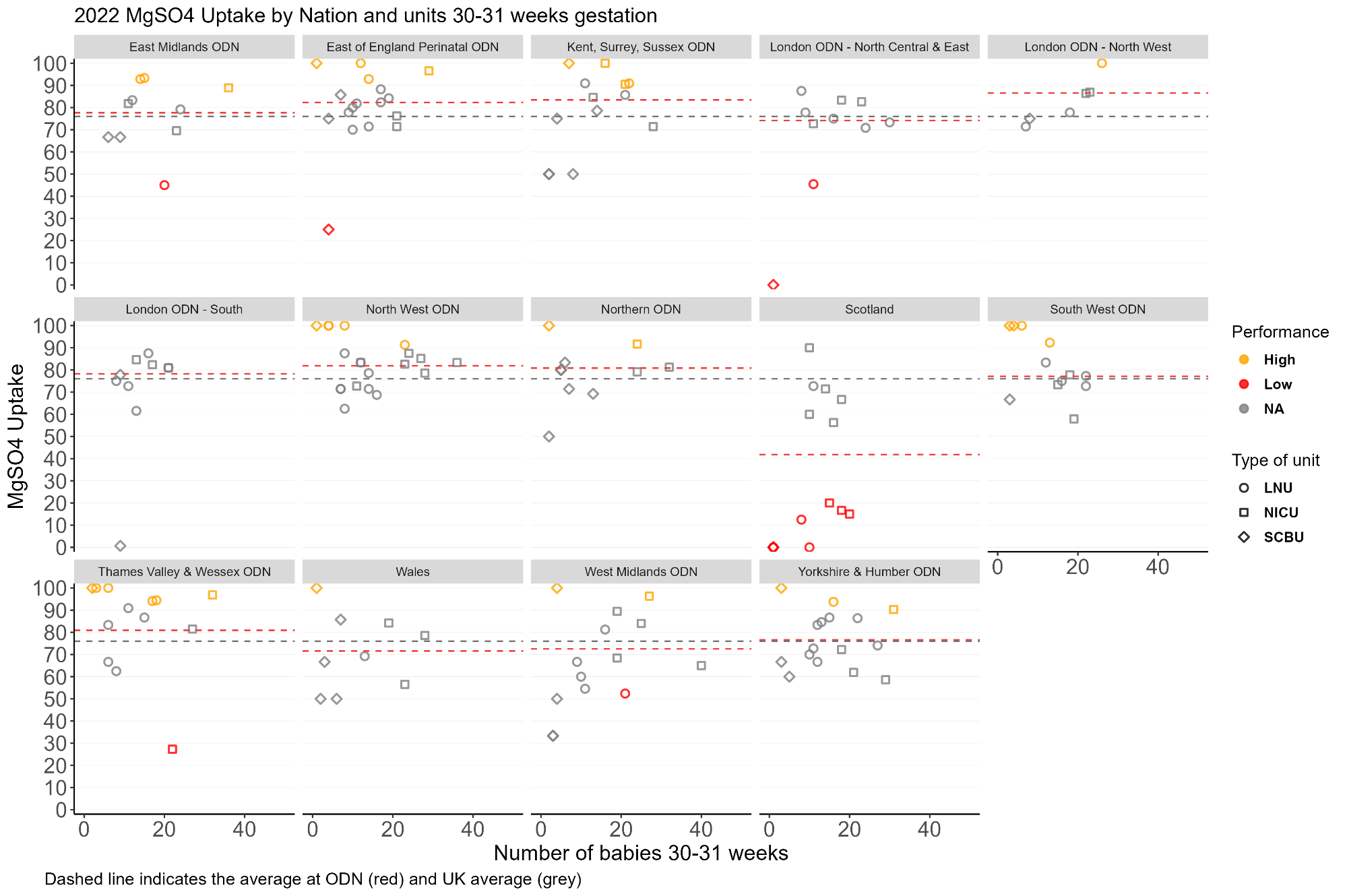


**Appendix F.** Net Monetary Benefit of MgSO_4_ implementation in 2014 and 2022 for England, Scotland and Wales – Optimal implementation considered at 90%.

| **Gestation weeks** | **Dimensions** | **England** | | **Scotland** | | **Wales** | |
| --- | --- | --- | --- | --- | --- | --- | --- |
|  |  | **2014** | **2022** | **2014** | **2022** | **2014** | **2022** |
| Less than 30 | Number of babies, N | 4,003 | 3,744 | 237 | 292 | 160 | 152 |
|  | Uptake of MgSO_4_, % | 36% | 85% | 39% | 82% | 20% | 86% |
|  | INMB of optimal MgSO_4_ implementation (90%), £ | 86,169,379 | 80,594,093 | 5,101,709 | 6,285,650 | 3,444,192 | 3,271,982 |
|  | INMB of actual implementation, £ | 34,293,919 | 76,530,120 | 2,205,233 | 5,705,205 | 735,248 | 3,152,670 |
|  | INMB forgone due to sub-optimal implementation (90%), £ | 51,875,460 | 4,063,973 | 2,896,477 | 655,026 | 2,708,944 | 119,313 |
| Between 30 and under 32 | Number of babies, N | 3,042 | 2,553 | 139 | 215 | 134 | 117 |
|  | Uptake of MgSO_4_, % | 19% | 79% | 19% | 42% | 13% | 72% |
|  | INMB of optimal MgSO_4_ implementation (90%), £ | 65,482,700 | 54,956,389 | 2,992,142 | 4,628,133 | 2,884,511 | 2,518,565 |
|  | INMB of actual implementation, £ | 14,072,670 | 48,260,861 | 673,111 | 2,148,588 | 431,188 | 2,005,685 |
|  | INMB forgone due to sub-optimal implementation (90%), £ | 51,410,030 | 6,695,527 | 2,319,031 | 2,479,545 | 2,453,322 | 512,881 |
| **Less than 32 (Total)** | **Number of babies, N** | 7,045 | 6,297 | 376 | 507 | 294 | 269 |
|  | **Uptake** **MgSO_4_, %** | 28% | 82% | 29% | 62% | 17% | 79% |
|  | **INMB of optimal MgSO_4_ implementation (90%), £** | 151,652,079 | 135,550,481 | 8,093,851 | 10,913,783 | 6,328,703 | 5,790,548 |
|  | **INMB of actual implementation, £** | 48,366,589 | 124,790,981 | 2,878,343 | 7,853,794 | 1,166,436 | 5,158,354 |
|  | **INMB forgone due to sub-optimal implementation (90%), £** | 103,285,490 | 10,759,500 | 5,215,508 | 3,134,570 | 5,162,266 | 632,193 |

MgSO_4_ – Magnesium sulphate

INMB – Incremental Net Monetary Benefit, estimated at a willingness-to-pay threshold of £20,000 per Quality Adjusted Life Year

**Appendix G.** High-cost scenario - National Value of Implementation for potential initiatives to Increase MgSO_4_ uptake in England, Scotland and Wales with three different implementation effectiveness and implementation costs for a single year (under 32 weeks gestation).

|  | **England** | | **Scotland** | | **Wales** | | **Total** | |
| --- | --- | --- | --- | --- | --- | --- | --- | --- |
| **Number of babies, N** | **6,297** | | **507** | | **269** | | **7,073** | |
| **Low performance: Implementation effect: 1%** | |  | |  | |  | |  |
| Increment of pre-term babies treated with MgSO_4_ | 63 | | 5 | | 3 | | 71 | |
| Net cost of implementation, £ | 1,677,000 | | 189,000 | | 109,000 | | 1,975,000 | |
| Implementation cost-effectiveness, £ per additional patient treated | 26,632 | | 37,278 | | 40,520 | | 27,923 | |
| **Net Monetary Benefit of the Policy^*^, £** | **-170,829**  **(-640,364; 362,999)** | | **-67,731**  **(-105,536; -24,750)** | | **-44,658**  **(-64,716; -21,854)** | | **-283,219**  **(-810,616; 316,395)** | |
| **Mid performance: Implementation effect: 5%** | |  | |  | |  | |  |
| Increment of pre-term babies treated with MgSO_4_ | 315 | | 25 | | 13 | | 354 | |
| Net cost of implementation, £ | 1,677,000 | | 189,000 | | 109,000 | | 1,975,000 | |
| Implementation cost-effectiveness, £ per additional patient treated | 5,326 | | 7,456 | | 8,104 | | 5,585 | |
| **Net Monetary Benefit of the Policy^*^, £** | **5,853,853**  **(3,535,570; 8,446,193)** | | **417,343**  **(230,688; 626,064)** | | **212,709**  **(113,674; 323,450)** | | **6,483,905**  **(3,879,932; 9,395,707)** | |
| **High performance: Implementation effect: 10%** | |  | |  | |  | |  |
| Increment of pre-term babies treated with MgSO_4_ | 630 | | 51 | | 27 | | 707 | |
| Net cost of implementation, £ | 1,677,000 | | 189,000 | | 109,000 | | 1,975,000 | |
| Implementation cost-effectiveness, £ per additional patient treated | 2,663 | | 3,728 | | 4,052 | | 2,792 | |
| **Net Monetary Benefit of the Policy^*^, £** | **13,384,706**  **(8,748,139; 18,569,385)** | | **1,023,686**  **(650,375; 1,441,128)** | | **534,417**  **(336,349; 755,900)** | | **14,942,810**  **(9,734,864; 20,766,414)** | |
